# Color Stability of Extrinsically Colored Monolithic Zirconia: A Narrative Review

**DOI:** 10.7759/cureus.106225

**Published:** 2026-03-31

**Authors:** Nouhaila Mazzir, Ichraq Benazouz, Imane Hachami, El Mehdi Jouhadi

**Affiliations:** 1 Department of Prosthodontics, Faculty of Dental Medicine, Hassan II University, Casablanca, MAR; 2 Department of Biology and Basic Subjects, Faculty of Dental Medicine, Hassan II University, Casablanca, MAR; 3 Department of Prosthodontics, Centre de Consultations et de Traitements Dentaires (CCTD), Ibn Rochd University Hospital Center, Casablanca, MAR; 4 Department of Fixed Prosthodontics, Faculty of Dental Medicine, Hassan II University, Casablanca, MAR

**Keywords:** ceramic, color, materials testing, prosthesis coloring, spectrophotometry, zirconium

## Abstract

Monolithic zirconia has become a widely used restorative material in prosthodontics due to its high mechanical strength, biocompatibility, and improved esthetic properties. To further enhance esthetic outcomes, extrinsic staining is commonly applied to monolithic zirconia restorations, allowing individualized shade characterization and surface effects. However, because these coloring agents are in a superficial layer, concerns remain regarding their long-term durability and color stability under different oral conditions. This narrative review aimed to evaluate the long-term color stability and optical durability of monolithic zirconia crowns treated with extrinsic stains, under simulated aging conditions. An electronic search was conducted across PubMed, ScienceDirect, and Wiley Online Library databases between 2016 and February 2026, investigating the effects of mechanical, chemical, thermal, and hydrothermal aging on extrinsically stained monolithic zirconia. A total of 1354 articles were initially identified. After removing duplicates and screening titles and abstracts, nine articles met all inclusion criteria and were included in the final analysis. These studies primarily evaluated color variation (ΔE, ΔE_00_), translucency-related parameters (relative translucency parameter, or RTP), and surface roughness (Ra) following simulated oral challenges. The included studies suggest that extrinsically stained monolithic zirconia demonstrated generally acceptable color stability, with most ΔE and ΔE_00_ values remaining within clinical thresholds. Mechanical surface alterations, particularly occlusal adjustments exceeding 100 μm, were identified as the most significant factor influencing discoloration. Chemical exposure to staining agents such as coffee produced moderate color changes, and while thermocycling alone had a limited impact, it affected translucency more than color stability. Extrinsically stained zirconia crowns retain satisfactory color stability and esthetic durability over time, provided that the glaze layer remains intact, and surface adjustments are limited. Their longevity depends more on clinical handling than on oral conditions.

## Introduction and background

With the evolution of ceramic materials, monolithic zirconia has become one of the essential restorative materials in modern prosthodontics due to its superior mechanical properties, biocompatibility, and esthetic potential [1]. Despite the esthetic improvements achieved with new generations of zirconia, there are situations where surface coloring may still be necessary to achieve optimal esthetic outcomes [2]. The coloring of zirconia restorations can be achieved either through extrinsic staining or by using precolored zirconia blocks or discs [3]. Extrinsic staining involves the application of coloring agents onto the surface of the restoration after sintering, followed by glazing or mechanical polishing. In contrast, precolored zirconia incorporates pigments within the material during manufacturing, and the restorations are subsequently finished directly by polishing or glazing after sintering [2]. The convenience of extrinsic staining comes with a trade-off: colorimetric effects are concentrated in a superficial layer that may be challenged by chemical oral conditions such as staining beverages and acidic drinks, hydrothermal and thermal conditions, and especially by mechanical wear or chairside adjustment that physically alters the surface. Therefore, it leads us to the question of the use of extrinsic staining in monolithic zirconia and the different parameters influencing the color stability [4,5]. In this context, narrative syntheses have also highlighted the role of surface finishing protocols (polishing versus glazing) in preserving the colorimetric stability of colored monolithic zirconia [6]. Color stability is usually expressed as ΔE*_ab_ (CIELAB) or ΔE_00_ (CIEDE2000). CIEDE2000 is often preferred in dentistry because of improved perceptual uniformity [7,8]. For clinical interpretation, widely cited 50:50% perceptibility and acceptability thresholds for dental ceramics have been proposed. However, thresholds are context-dependent and should be applied cautiously as interpretive aids rather than strict pass/fail rules [9]. Accordingly, a numerical comparison between ΔE*_ab_ and ΔE_00_ was intentionally avoided throughout this narrative synthesis because these metrics are not interchangeable.

This narrative review was conducted to synthesize current scientific data evaluating the color stability of extrinsically stained monolithic zirconia crowns under different oral conditions. This was achieved through an examination of the effects of chemical, mechanical, thermal, and hydrothermal aging conditions on the color stability and translucency of restorations interpreted in various studies.

## Review

Methodology

This study was conducted as a narrative review aimed at exploring and synthesizing contemporary scientific evidence regarding the color stability and optical durability of extrinsically stained monolithic zirconia. A structured electronic literature search was performed across PubMed, ScienceDirect, and Wiley Online Library databases between 2016 and February 2026. Publications addressing color stability, translucency behavior, and aging response of monolithic zirconia treated with extrinsic staining were considered. Searches used combinations of keywords and MeSH terms including “Color”, “Ceramic”, “Materials Testing”, “Zirconium’’, “Dental Restoration, Permanent”, “Prosthesis Coloring”, “Spectrophotometry” using Boolean equation, as follows: (zirconia OR "monolithic zirconia" OR "zirconium oxide") AND (color OR "color stability" OR discoloration OR "color change" OR translucen* OR "optical propert*") AND (dental OR ceramic* OR restor* OR prosthodont*).

**Figure 1:**
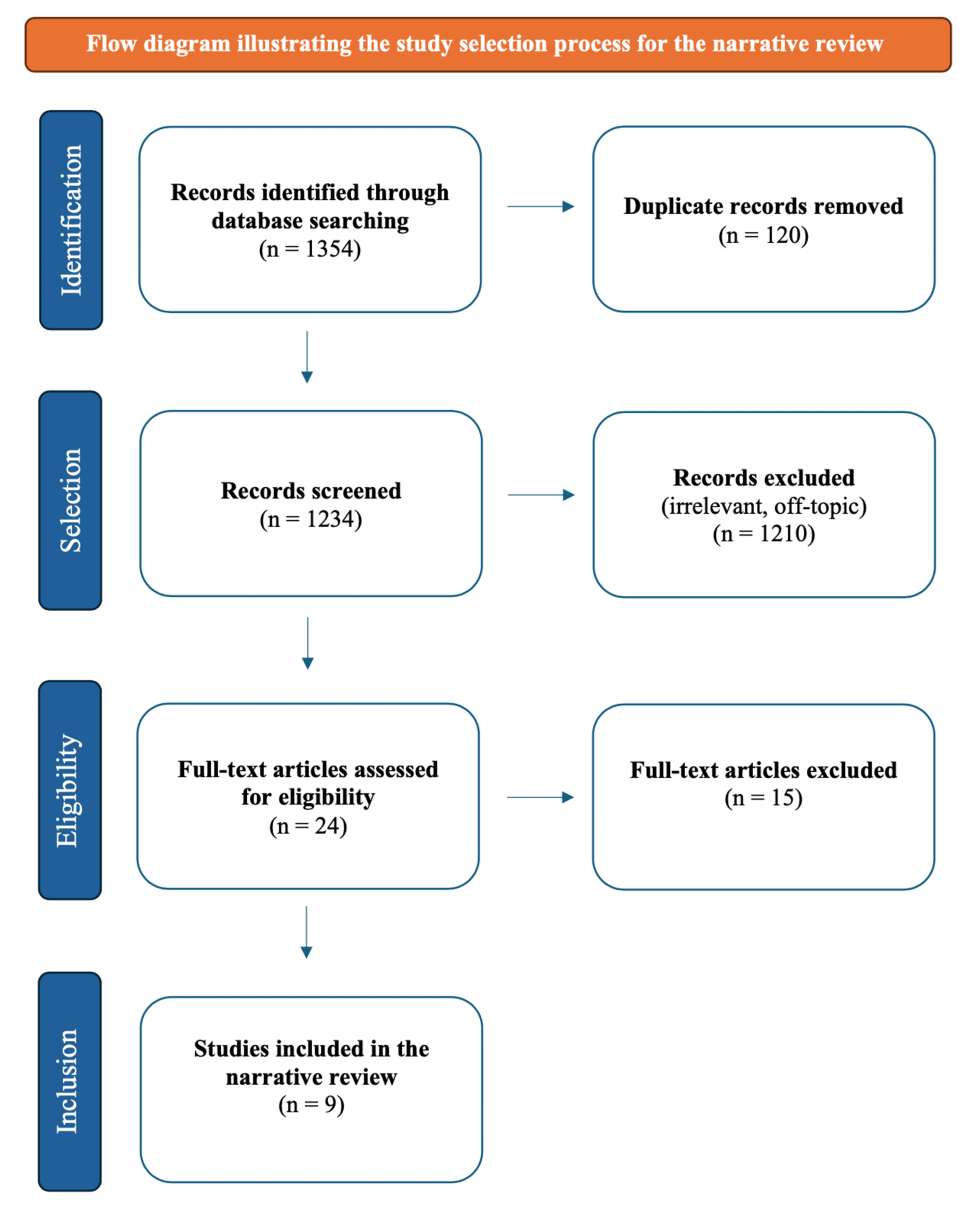
Flow diagram illustrating the study selection process for the narrative review

Two independent reviewers assessed the studies identified through the search strategy. During the initial screening phase, titles and abstracts were carefully examined to determine their relevance in accordance with the study objectives. Full-text articles were subsequently evaluated based on predefined inclusion and exclusion criteria, and only studies meeting all eligibility requirements were selected for final inclusion. The inclusion criteria were as follows: studies assessing color stability and translucency of extrinsically stained zirconia, studies evaluating optical modifications following simulated aging conditions, studies published in English between 2016 and February 2026, and studies limited to zirconia materials. Studies focusing exclusively on precolored (intrinsically pigmented) zirconia, studies lacking simulated aging or colorimetric evaluation, and non-peer-reviewed sources or abstract-only publications were excluded. Any disagreements between the two reviewers were resolved through discussion and consultation with a third reviewer. Ultimately, nine studies fulfilled the eligibility criteria following full-text assessment. The study selection process, from initial database identification to final inclusion of eligible studies, is summarized in the flow diagram presented in Figure 1.

Table 1 provides an overview of the included studies and the aging protocols evaluated.

**Table 1:** Overview of the studies selected for the narrative review

Author(s)	Year	Study Type	Study Title	Type of Aging/Exposure
Spyropoulou et al. [3]	2016	In vitro study	Cyclic loading effect on color stability of unshaded versus shaded zirconia	Mechanical aging (cyclic mechanical loading)
Papageorgiou-Kyrana et al. [5]	2018	In vitro study	Evaluation of color stability of preshaded and liquid-shaded monolithic zirconia	Thermal aging (thermocycling)
Subaşı et al. [10]	2018	In vitro study	Effects of fabrication and shading technique on the color and translucency of new-generation translucent zirconia after coffee thermocycling	Chemical and thermal aging (coffee thermocycling)
Alp et al. [11]	2018	In vitro study	Effect of shading technique and thickness on color stability and translucency of new generation translucent zirconia	Chemical and thermal aging (coffee thermocycling)
Rafael et al. [12]	2018	In vitro study	Impact of laboratory treatment with coloring and fluorescent liquids on the optical properties of zirconia before and after accelerated aging	Accelerated hydrothermal aging (autoclave)
Herpel et al. [4]	2021	In vitro study	Color stability of individually stained monolithic zirconia following occlusal adjustment	Mechanical aging
Kanat-Ertürk [6]	2020	In vitro study	Color stability of CAD/CAM ceramics prepared with different surface finishing procedures	Chemical aging (coffee and tea)
Bayestehtarat et al. [13]	2023	In vitro study	Longevity of extrinsic stains on monolithic zirconia restorations: an in vitro study	Thermal and mechanical aging (thermocycling and toothbrushing)
Abounassif et al. [14]	2026	In vitro study	Color stability of precolored and extrinsically colored monolithic multilayered polychromatic zirconia: Effects of surface finishing and aging	Mechanical and thermal aging (thermocycling and simulated toothbrushing)

Results

From the literature search across various databases, the initial identification process revealed 1354 research papers. The final phase led to nine research papers being selected for qualitative and quantitative analysis, while 15 additional research papers were excluded for various reasons.

The reviewed literature reveals a structured body of evidence investigating the color and optical stability of extrinsically stained monolithic zirconia under simulated oral aging conditions. This artificial aging process incorporated four different techniques that included hydrothermal methods (autoclave simulation), thermal aging (thermocycling protocols), chemical aging (immersion in staining media such as coffee, tea, alcoholic beverages, or mouthrinses), and mechanical aging (cyclic loading, occlusal adjustment, and simulated toothbrushing).

Thermal aging was investigated in five studies, making it the most frequently evaluated aging protocol [5,10,11,13,14], whereas hydrothermal aging was assessed in only one study [12]. Two of the five studies combined thermal aging with mechanical aging [13,14], and two studies also associated thermal aging with chemical aging [10,11]. Overall, the results showed that thermal aging had a limited impact on color stability, with ΔE and ΔE_00_ values generally remaining below clinically acceptable thresholds. However, translucency-related parameters appeared to be more sensitive to thermal challenges than color difference values.

Chemical aging was assessed in three studies [6,10,11]. Two of these investigations combined chemical and thermal aging through coffee thermocycling [10,11], which primarily affected translucency rather than color stability. Conversely, the study evaluating immersion in coffee or tea alone reported preserved color stability when a glazing finishing procedure was applied [8].

Mechanical aging was also investigated in three studies and had the most impact on color stability of all the artificial aging techniques [3,4,13]. A significant relationship was proven between mechanical removal and color stability of individually stained zirconia; the threshold of acceptability of color differences in zirconia was exceeded (ΔE_00_ >1.8) in a linear manner when mechanical removal exceeded 100 μm.

Across the included studies, color stability was predominantly evaluated using ΔE (CIELAB) and ΔE_00_ (CIEDE2000) color-difference formulas. Some studies also evaluated the relative translucency parameter (RTP) to see the impact of artificial aging on the translucency of stained zirconia [10,11], and only one study evaluated the impact on surface roughness (Ra) [12].

Most studies reported that values remained within clinically acceptable thresholds (ΔE ≤ 3.3-3.7 and ΔE_00_ ≤ 1.8) [3,5,6,11,14], indicating generally favorable color stability, particularly when it was associated with a glaze surface finishing procedure [6,14]. The most pronounced color changes were observed after mechanical adjustments [4], whereas chemical exposure induced moderate discoloration, but was still clinically acceptable in most studies [6,10,11]. In contrast, thermal aging showed minimal impact on color stability but significantly affected translucency [10,11]. Table 2 provides a structured summary of the main findings and methodological characteristics of all analyzed studies. A thorough analysis is addressed in the Discussion section.

**Table 2:** A structured summary of the main findings and methodological characteristics of all analyzed studies ΔE_00_ = CIEDE2000 color difference; RTP = relative translucency parameter; ΔE = CIELAB color difference; Ra = arithmetic mean roughness

S. No.	Author(s)	Year	Aging Method	Parameter Measured	Main Findings
1	Herpel et al. [4]	2021	Mechanical aging: instant surface reduction (20-500 μm)	ΔE_00_	Occlusal adjustment exceeding 100 μm resulted in color changes surpassing the clinical acceptability threshold.
2	Abounassif et al. [14]	2026	Thermocycling (5,000 cycles) + simulated toothbrushing	ΔE_00_	6Y-PSZ is less stable than 3Y-TZP; external staining is stable with glazing and less stable with polishing; precolored zirconia is stable with both surface polishing and glazing.
3	Spyropoulou et al. [3]	2016	Cyclic mechanical loading (500,000 cycles)	ΔE	L* a* b* values for both shaded and unshaded zirconia were affected by cyclic loading, but still clinically acceptable ΔE < 3.7.
4	Subaşı et al. [10]	2018	Coffee thermocycling 10,000 cycles	ΔE_00_, RTP	Externally stained zirconia had a greater color change than preshaded, either in monolithic or veneered form; fabrication technique affected the RTP, and the monolithic zirconia showed more translucency than the veneered ones.
5	Alp et al. [11]	2018	Coffee thermocycling 10,000 cycles	ΔE_00_, RTP	ΔE_00_ was similar across groups; both preshaded and externally shaded were not affected by the thermocycling. RTP values were affected; externally shaded zirconia presented higher RTP than preshaded zirconia. Translucency also decreases with increased thickness.
6	Papageorgiou-Kyrana et al. [5]	2018	Thermocycling (number of cycles not reported)	ΔE	All ΔE < 3.7; both preshaded and liquid-shaded monolithic zirconia were not affected by thermocycling; liquid-shaded zirconia was slightly more stable.
7	Rafael et al. [12]	2018	Accelerated hydrothermal aging; autoclave (1-5 h)	ΔE_00_, fluorescence	Accelerated aging significantly increases discoloration, especially in fluorescent-treated zirconia.
8	Kanat-Ertürk [6]	2020	Chemical aging in coffee and tea (2 months)	ΔE	Glaze procedure led to more color stability compared to mechanical polishing and external staining with glaze for both zirconia-reinforced lithium silicate ceramic and lithium disilicate glass ceramic.
9	Bayestehtarat et al. [13]	2023	Thermocycling (10,000 cycles) and toothbrushing (1 year)	ΔE, Ra	Monolithic zirconia showed changes in color and surface roughness that did not surpass the thresholds for perceptibility and acceptability.

Discussion

Extrinsically stained monolithic zirconia is increasingly used in dental restorations due to its superior esthetic qualities and mechanical properties. However, its long-term performance under various artificial aging conditions remains a critical determinant of clinical success. Changes in color and optical behavior have been assessed using different aging protocols, including hydrothermal, thermal, chemical, and mechanical aging, which simulate the clinical and environmental conditions that zirconia restorations may encounter over time. Understanding these effects is essential for predicting the longevity and reliability of extrinsically stained monolithic zirconia restorations in clinical practice. Next, we provide an in-depth analysis of these different factors and their clinical implications.

Mechanical Aging

Among all simulated aging conditions, mechanical intervention emerged as the most critical determinant of color instability in extrinsically stained monolithic zirconia. Across the included studies, mechanical surface reduction consistently produced greater color alterations compared with chemical or thermal aging alone [3,4,11,14].

Herpel et al. demonstrated that occlusal adjustment exceeding 100 μm resulted in ΔE_00_ values surpassing the 50:50% acceptability threshold, indicating clinically perceptible discoloration. This finding highlights the superficial localization of extrinsic pigments, which are confined to a thin characterization layer vulnerable to grinding or polishing procedures. Once this layer is partially removed, the underlying zirconia substrate becomes exposed, leading to measurable color deviation [4]. Similarly, cyclic mechanical loading was shown to influence L*, a*, and b* parameters, although changes generally remained within clinically acceptable limits [3]. These findings suggest that progressive surface degradation is responsible for color alterations rather than intrinsic material instability.

The influence of surface finishing further reinforces this interpretation. Studies comparing glazing and polishing protocols consistently demonstrated that glazed specimens exhibited superior color stability following mechanical challenges or even chemical conditions [6,14]. The glaze layer appears to act as a protective barrier, preserving pigment integrity and minimizing surface roughness, whereas polished surfaces are more susceptible to pigment loss and optical alteration [2].

Additionally, zirconia composition plays a modulatory role. Highly translucent 6Y-PSZ zirconia was found to exhibit lower color stability compared with 3Y-TZP under combined mechanical and thermal aging, possibly due to differences in microstructure and phase composition that influence optical scattering and surface behavior [2].

These findings align with comparative studies, such as the one by Al-Zordk and Saker, who showed that clinical adjustments combined with thermocycling create measurable color change but still clinically acceptable ΔE values, emphasizing that the severity of mechanical reduction determines the extent of color change [15]. Collectively, these findings indicate that mechanical disruption of the superficial stain layer constitutes the primary mechanism of discoloration in extrinsically characterized zirconia. From a clinical perspective, minimizing occlusal adjustment and preserving the glaze layer are essential strategies for maintaining long-term esthetic stability [4].

Chemical Aging

Chemical aging is also one of the most widely explored aging methods in the literature. However, most studies have focused on the impact of chemical aging on zirconia as a bulk substrate, and only a few have examined the chemical aging impact on extrinsically stained zirconia surfaces. In the present synthesis, studies evaluating chemically induced aging of extrinsically characterized zirconia predominantly relied on immersion in coffee and tea, occasionally combined with thermocycling [6,10,11]. Collectively, these investigations demonstrate that chemical exposure alone induces measurable but generally clinically acceptable color alterations [6,10,11].

Color stability appears to be more influenced by surface finish and the shading technique than by the oral environment itself [10,14]. Externally shaded zirconia exhibited greater discoloration compared with preshaded zirconia after coffee thermocycling, supporting the concept that intrinsic pigmentation distributed throughout the material provides greater long-term stability than superficial characterization. However, in both groups, the color changes remained imperceptible and within clinically acceptable limits [10]. In particular, glazing consistently emerged as the most protective factor, significantly reducing color change compared with polished surfaces, with most ΔE values remaining within the clinically acceptable threshold [6].

An additional pattern observed across studies is that chemical aging tends to affect translucency parameters more than color stability itself, particularly when combined with thermocycling [10,11].

In fact, Alp et al. investigated the effect of shading technique and material thickness on the color stability of translucent zirconia after coffee thermocycling. Their findings demonstrated that neither shading technique nor thickness significantly affected color stability; it indeed significantly affected the RTP, indicating that optical behavior may change without clinically perceptible color alteration [11].

When zirconia was compared with hybrid or glass-based ceramics, it consistently exhibited superior resistance to staining beverages and acidic environments such as mouthrinses, coffee, tea, and cola [16,17]. These findings reinforce the concept that discoloration following chemical exposure primarily originates from alterations of the extrinsic stain layer rather than degradation of the zirconia core material.

In agreement with these findings, Colombo et al. reported that alcoholic mouthrinses and acidic beverages induced only minimal color variations (ΔE ≤ 2.8) in zirconia, reinforcing its intrinsic chemical resistance [18]. However, Arindham et al. [19] demonstrated that immersion in alcoholic beverages such as red wine and whiskey not only produced moderate ΔE changes but also significantly increased surface roughness. These findings suggest that surface roughness may act as a facilitating factor for pigment retention and optical alteration.

Consequently, the protective effect observed in glazed specimens across multiple studies can be partially explained by the smoother surface topography provided by the glaze layer, which limits stain adherence and surface degradation [6,14]. Across studies, the pattern is clear: chemical exposure leads to moderate discoloration, but it is still clinically acceptable. Zirconia remains among the most stable restorative materials in such conditions compared to hybrid and feldspathic ceramics. Nevertheless, color stability is strongly influenced by coloring technique and principally surface finishing procedures.

Thermal and Hydrothermal Aging

Thermal aging through thermocycling was one of the most frequently investigated protocols among the included studies [5,10,11,13,14]. Overall, thermocycling alone demonstrated limited impact on color stability, with ΔE and ΔE_00_ values generally remaining below clinically acceptable thresholds across different zirconia types and shading techniques [5,11,14]. These findings suggest that temperature fluctuations and moisture exposure, when applied independently, do not significantly compromise the intrinsic optical stability of zirconia or its superficial characterization layer.

However, translucency-related parameters were more sensitive to thermal challenges than color difference values. Several investigations reported measurable reductions in the RTP, particularly in externally shaded and thinner specimens [10,11]. This indicates that thermal stress may alter light-scattering behavior without necessarily inducing clinically perceptible discoloration. In contrast, accelerated hydrothermal aging through autoclave simulation demonstrated a more pronounced effect on optical properties. Rafael et al. reported that hydrothermal exposure significantly increased discoloration, especially in zirconia treated with fluorescent characterization liquids, suggesting that certain surface additives may be more susceptible to degradation under combined heat and moisture conditions [12].

Importantly, when thermocycling was combined with mechanical or chemical challenges, greater color alterations were observed [11,14], reinforcing the fact that aging factors act synergistically rather than as isolated effects. Therefore, while thermal aging alone appears relatively benign, its interaction with surface treatments and mechanical wear may amplify optical instability.

Overall, temperature and humidity fluctuations do not constitute the primary cause of discoloration in extrinsically stained zirconia; rather, vulnerability arises when thermal stress acts in conjunction with surface modification or pigment-related variables [10,11,13,14].

Overall interpretation and clinical relevance/recommendations

Overall, the findings suggest that monolithic zirconia remains a robust restorative material, but the addition of extrinsic stains creates a layer of vulnerability. The collective evidence suggests several practical recommendations and therefore clinicians should consider the following: minimizing occlusal adjustment (<100 μm) on stained zirconia to avoid removing the stain layer, using glazing to protect extrinsic stains and reduce surface roughness and stain retention, preferring preshaded zirconia when mechanical adjustments or long-term stability are priorities, and anticipating moderate color changes in patients with high consumption of coffee, tea, and alcoholic beverages like red wine, and use of mouthrinses.

Limitations of the review

This narrative review presents several limitations that should be considered when interpreting the findings. First, all included studies were in vitro, which limits the ability to extrapolate results directly to clinical performance, since oral conditions involve dynamic interactions (saliva flow, biofilm formation, masticatory cycles) that cannot be completely simulated. Second, there was substantial heterogeneity in the aging protocols: immersion times varied from hours to weeks, while thermocycling ranged from 5,000 to 10,000 cycles, limiting direct comparison across studies. Third, the characteristics of the zirconia samples differed in terms of yttria content (3Y, 4Y, 5Y), translucency, manufacturing technique, and staining protocols, which introduces variability in outcomes. Furthermore, most studies did not report standardized ΔE_00_ thresholds or uniform measurement conditions (light source, spectrophotometer specifications), which impacts consistency. Finally, relatively few studies evaluated the combined effects of simultaneous chemical, thermal, and mechanical challenges, despite such combinations being more representative of real clinical conditions.

Future research directions

Future investigations should focus on standardizing aging protocols, including consistent thermocycling cycles, immersion times, and ΔE_00_ evaluation thresholds, to improve comparability across studies. Long-term clinical trials are needed to validate in vitro findings under real oral conditions. Finally, exploring innovative stain formulations and surface protection treatments may improve the durability of extrinsic characterization on monolithic zirconia.

## Conclusions

Extrinsically stained monolithic zirconia shows good overall color and optical stability, but its performance depends heavily on the aging environment and the integrity of the superficial stain layer. Mechanical adjustments were found to produce the greatest color changes, followed by chemical exposure that leads to moderate discoloration, but is still clinically acceptable. Finally, thermal aging had a limited effect on color stability but a greater effect on translucency. Clinically, minimizing surface reduction and protecting the stain layer through glazing are essential for maintaining long-term esthetics of extrinsically stained monolithic zirconia. Alternatively, precolored zirconia showed better color stability with both mechanical polishing and glazing.
